# Serotype-Specific Transmission and Waning Immunity of Endemic Foot-and-Mouth Disease Virus in Cameroon

**DOI:** 10.1371/journal.pone.0136642

**Published:** 2015-09-01

**Authors:** Laura W. Pomeroy, Ottar N. Bjørnstad, Hyeyoung Kim, Simon Dickmu Jumbo, Souley Abdoulkadiri, Rebecca Garabed

**Affiliations:** 1 Department of Veterinary Preventive Medicine, Ohio State University, Columbus, OH, United States of America; 2 Department of Biology, Pennsylvania State University, University Park, PA, United States of America; 3 Department of Entomology, Pennsylvania State University, University Park, PA, United States of America; 4 Center for Infectious Disease Dynamics, The Pennsylvania State University, University Park, Pennsylvania, United States of America; 5 Fogarty International Center, National Institutes of Health, Bethesda, Maryland, United States of America; 6 Department of Geography, Ohio State University, Columbus, OH, United States of America; 7 Laboratoire National Veterinaire, Lanavet, Garoua, Cameroon; 8 Public Health Preparedness for Infectious Disease Program, The Ohio State University, Columbus, OH, United States of America; Institut National de la Santé et de la Recherche Médicale (INSERM), FRANCE

## Abstract

Foot-and-mouth disease virus (FMDV) causes morbidity and mortality in a range of animals and threatens local economies by acting as a barrier to international trade. The outbreak in the United Kingdom in 2001 that cost billions to control highlighted the risk that the pathogen poses to agriculture. In response, several mathematical models have been developed to parameterize and predict both transmission dynamics and optimal disease control. However, a lack of understanding of the multi-strain etiology prevents characterization of multi-strain dynamics. Here, we use data from FMDV serology in an endemic setting to probe strain-specific transmission and immunodynamics. Five serotypes of FMDV affect cattle in the Far North Region of Cameroon. We fit both catalytic and reverse catalytic models to serological data to estimate the force of infection and the rate of waning immunity, and to detect periods of sustained transmission. For serotypes SAT2, SAT3, and type A, a model assuming life-long immunity fit better. For serotypes SAT1 and type O, the better-fit model suggests that immunity may wane over time. Our analysis further indicates that type O has the greatest force of infection and the longest duration of immunity. Estimates for the force of infection were time-varying and indicated that serotypes SAT1 and O displayed endemic dynamics, serotype A displayed epidemic dynamics, and SAT2 and SAT3 did not sustain local chains of transmission. Since these results were obtained from the same population at the same time, they highlight important differences in transmission specific to each serotype. They also show that immunity wanes at rates specific to each serotype, which influences patterns of local persistence. Overall, this work shows that viral serotypes can differ significantly in their epidemiological and immunological characteristics. Patterns and processes that drive transmission in endemic settings must consider complex viral dynamics for accurate representation and interpretation.

## Introduction

Foot-and-mouth disease virus (FMDV) is currently ranked as one of the most significant foreign animal disease threats in most major global economies [1, 2]. Perhaps surprisingly, this is not because the virus causes exceptionally severe disease or because there is no preventative vaccine; in fact, morbidity is moderate, disease-induced mortality is only slightly elevated in animals up to one year of age [1], and vaccines exist against multiple FMDV serotypes [3, 4]. The high threat raking is due, at least partially, to the intersection between epidemiology and economics. The World Organisation for Animal Health (OIE) classifies countries into three epidemiological categories with regards to foot-and-mouth (FMD) disease status: countries with active FMDV outbreaks, countries that are free of disease in the presence of vaccination, and countries that are free of the disease in the absence of vaccination [5]. Countries experiencing active FMDV outbreaks have the most restricted export market, while countries that are FMDV free without vaccination have the most accessible export market. Following the outbreak of FMD in the UK in 2001, which cost £4 billion to successfully eliminate the Pan-Asia O serotype [6], computational efforts have been initiated in many countries that are free of FMDV in the absence of vaccination like the United States [7–10], Australia [11, 12], and throughout Europe [13–15]. These projects analyze threats and plan disease management strategies if an epidemic of any of the multiple circulating FMDV serotypes were to be introduced.

We contend that for any of these efforts to be successful in making robust predictions about future epidemic scenarios, we need to understand the epidemiology of FMDV in much greater detail by quantifying strain-specific transmission and immunodynamics in the endemic setting. A quantity of particular interest is the force of infection (FOI), which measures the rate at which susceptible individuals acquire infection. The FOI can inform epidemic behavior, identify heterogeneities in transmission, and specify targets for disease control [16–19]. Standard approaches to estimating the FOI fit accurate counts of both susceptible and infected individuals to dynamical catalytic models. However, this information is difficult to obtain, especially for endemic disease when there is little or inconsistent surveillance, or when the disease can be subclinical. In these cases, the lack of necessary information prohibits the design and implementation of disease control programs.

One solution to quantifying disease dynamics in the absence of counts of infected and susceptible individuals is to fit dynamical models to serology data. These data classify an individual based on the presence of host antibodies against a pathogen, which indicates past exposure to the pathogen. Here, we show that serology data matched with host age can be fitted to dynamical models to provide a record of historical exposure, even when animals are sampled only in a single event such as a cross-sectional study. The serology data also allow us to estimate the duration of immunity for different strains—an important epidemiological parameter that is poorly known for FMD. We use serology data that indicate past exposure to FMDV for cattle in the Far North Region, Cameroon, where there is evidence that five of the seven serotypes are present either endemically or intermittently [20].

Approximately 650,000 cattle are herded in the Far North Region, Cameroon [21]. Herd management is highly individual and non-intensive, but can be broadly classified in one of two ways. The majority of cattle herds (~93%) are sedentary, residing near villages and grazing around them. The minority of cattle herds (~7%) are mobile, participating in transhumance which allows them to graze on pastures in different geographic areas throughout the year depending on seasonal availability. Wild bovids are absent from this site. Herders report frequent FMD outbreaks; however, there was a complete absence of formal control and no access to FMDV vaccines at the time of the study [20, 22].

To address uncertainty in the duration of a host immune response against FMDV in the absence of vaccination, we fit contrasting models of FMDV transmission and immunity to the serology data: the catalytic model and reversible catalytic model [23, 24]. The catalytic model describes transmission followed by lifelong seroconversion, suggesting that the antibody response lasts for the lifespan of the animal. Alternatively, the reversible catalytic model assumes that infection leads to temporary seroconversion, and that the immune memory wanes over time. We also discriminate between two hypotheses about variation in the FOI. Previous studies have shown that the FOI is age-specific, due to the fact that animals have contact rates that vary with age, in an endemic steady state [16, 25]. Other studies have also shown that the FOI may be time-varying, due to the fact that incidence often exhibits cycles or local extinction due to depletion of susceptible individuals which do not support the idea of a disease steady state [17]. We distinguish between age-specific and time-varying differences in the force of infection by estimating the force of infection for five FMDV serotypes independent of each other and then comparing results across serotypes. In this way, we test between two sets of alternative hypothesizes: first, between lifelong seroconversion versus waning immunity and second, between age-specific FOI versus time-varying FOI.

Within this framework, we characterize and quantify three aspects of FMDV dynamics in the endemic setting. First, we determine the duration of seropositivity as a result of natural infection in the absence of vaccination. Second, we determine if the FOI is age-specific or time-varying and quantify the FOI for each serotype. Third, we use the FOI patterns inferred to estimate key epidemiological quantities that describe endemic FMDV dynamics. By estimating these key epidemiological parameters, we are able to reconstruct the historical burden of FMDV in an endemic area and quantify control efforts necessary to stop transmission.

## Materials and Methods

### Data collection

Data were obtained in four phases from cattle over one year of age in the Far North Region, Cameroon between February 5, 2010 and December 29, 2010 and again between January 30, 2012 and February 8, 2012. The cattle were from 15 mobile herds, 15 sedentary herds. First, cattle ages were obtained by interviews with herders. Second, serum samples were taken from the same 30 herds using a sampling scheme detailed in Ludi *et al*. [20]. The herds were visited twice during the study period and the same animals were located and sampled on subsequent visits whenever possible. In 2010, 531 samples were obtained from 498 animals. Four hundred sixty six animals were sampled once, thirty-one animals were sampled twice, and one animal was sampled three times during the study period (A Text in S1 File). In 2012, 124 samples were obtained. Animal ages collected in the first phase were confirmed upon sampling conducted in the second phase and the samples and age data were then linked by an animal identification number. Third, serum samples were shipped to the USA and subjected to serum neutralization tests (SNTs) at Plum Island Animal Diagnostic Center (PIADC), Agricultural Research Service (ARS), United States Department of Agriculture (USDA). Each serum sample was tested for serotypes O, A, SAT1, SAT2, and SAT3 as reported in Ludi *et al*. [20]. Fourth, the remainders of the serum samples were irradiated and subjected to antibody testing, in which animal autoantibody profiles were determined at the National Veterinary Services Laboratory (NVSL), USDA. This analysis confirmed the identities of serially sampled animals.

### Initial data analysis

A subset of animals (n = 32) were sampled two or three times in 2010. For this subset, the serology status of serially sampled animals was compared across sampling events, with an interval between sampling events that ranged from 94 to 190 days (A Text in S1 File). Paired samples taken from a single animal at two different time points were classified into one of four groups: animals that retained a positive serology status between the two sampling events; animals that had a positive serology status at the first sampling event and a negative serology status at the second sampling event; animals that had a negative serology status at the first sampling event and a positive serology status at the second sampling event; and animals that retained a negative serology status between the two sampling events. Paired samples were separately classified for each of the five FMDV serotypes. We used this information only as a preliminary investigation to determine plausibility of investigating the hypothesis of waning immunity for each serotype.

### Modeling transmission and immunity

We compared two models to determine which better fit the age and serology data collected in Cameroon. The first model was a catalytic model, which assumes that immunity is lifelong. The second model was a reverse catalytic model, which assumes that immunity is transient and wanes over time. We fit both models to the dataset of all animals (n = 498) sampled once, twice, and three times, which amounted to 531 samples and was much larger than the subset used in the initial data analysis. We selected the model that best fit the dataset of all animals using AIC. In this way, we could determine if lifelong or waning immunity best described immunological processes in our study population for each FMDV serotype.

To define the two models mathematically, let *a* represent the age of each animal in years and let *t* represent a calendar year. Let *λ*(*t*) represent the force of infection—the rate per capita per year at which seronegative individuals become seropositive due to infection. According to the catalytic model [16, 18, 23, 24, 26], assuming lifelong immunity, the age-specific seroprevalence is given by

$$P(a)=1-e^{-\int_{0}^{t}\lambda (t)\;dt}.$$ (1)

In our analysis, we can ignore any maternally derived antibodies since all animals were at least one year of age. We can use the reverse catalytic model to estimate the age-specific seroprevalence, given by

$$P(a)=\frac{\lambda (t)}{\lambda (t)+\omega }(1-e^{-\int_{0}^{t}(\lambda (t)+\omega )\;dt}),$$ (2)

assuming seroconversion lasts for an average duration of 1ω and then wanes [23, 24]. In both models, we accounted for gradual changes in the force of infection by fitting the force of infection as a b-spline that varied by year. The b-spline, fit with the *bs* command in the *splines* package in R, was a cubic spline with four uniformly spaced internal knots.

### Model selection, parameterization, and sensitivity

The catalytic and reverse catalytic models were compared and the best-fit model was selected using Akaike’s Information Criterion (AIC) [27]. To find estimates for the force of infection and rate of waning immunity, we estimated values for the b-splined force of infection and rate of waning immunity by maximizing the binomial likelihood of seropositivity by age according to

$$y_{a}\sim Bin\left(N_{a},p_{a}\right)$$ (3)

where *N*_*a*_ is the total number of animals sampled at age *a* and *p*_*a*_ is the proportion of animals that are seropositive. Likelihood estimations were performed using the *optim* command in R.

Plots of the time-varying forces of infection were compared across serotypes and local minima and maxima were visually determined. If local minima and maxima occurred at the same ages regardless of serotype, we can assume that the FOI is age-related and FMD dynamics are at or near a steady state. If local minima and maxima occurred at different ages across serotypes, we can assume that the FOI is time-varying and FMD dynamics are not at a steady state. (See C Text in S1 File for discussion.)

Ages were calculated from birth months and years reported in interviews. Therefore, the accuracy of reported ages may affect model selection and parameterization. We investigated the impact of inaccuracy in age reporting using the Morris Method for parameter sensitivity [28] to find the deviation from the reported age at which the model selected would change.

### Estimating key epidemiological quantities

The number of secondary cases produced by a primary infectious case, or *R*_*t*_, can be calculated from the parameterized FOI and the average life expectancy (*L*). Assuming Type I mortality, in which all animals live until a certain age and then die, the average life expectancy *L* = 16 years (see S3 Text in S1 File for discussion). It follows that we can estimate *R*_*t*_ from the *L* and the mean FOI ($\bar{\lambda }$), according to [29]

$$R_{t}=\bar{\lambda }L.$$ (4)

Assuming a fully efficacious vaccine, the critical proportion (*p*_*c*_) of the population that needs to be vaccinated to prevent an outbreak depends on *R*_*t*_ according to

$$p_{c}=1-\frac{1}{R_{t}}$$ (5)

All analyses were performed using R version 2.15.0 [30] in RStudio [31].

### Ethics statement

The animal use protocol for this study was reviewed and approved by The Ohio State University’s Animal Care and Use Program (Animal Welfare Assurance Number A3261-01). The protocol number in the Ohio State University system is 2010A0018, which superseded 2009A0113 (approval for the pilot project and first round of sampling in January and February 2010). The project was also approved through the Ministry of Scientific Research and Innovation of Cameroon with permit numbers 0000114/MINRESI/B00/C00/C10/C13 (pilot project and 2010 data) and 004/MINRESI/CRRI-EN/BAG/2011 (for 2011–2012). Herd owners formally consented to participation in the study at the beginning of the study. At each visit to the herd, herd owners and/or herders (whoever was present) assented to sampling of the animals and to responding to a survey, which included the age data presented here. Assent was recorded through use of a verbal consent script by the technicians collecting samples and administering the survey. The data used in this manuscript did not require IRB approval; though, the survey contained other information that did require IRB approval. The larger survey was approved by the Ohio State University Institutional Review Board/ Human Research Protection Program (Federal-wide Assurance #00006378 from the Office for Human Research Protections in the Department of Health and Human Services: protocol 2010B0004). Within this ethical review for the survey the protocol was approved for a waiver of signed consent forms due to the low literacy of the population and cultural inappropriateness of obtaining signatures to record consent.

## Results

**Fig 1:**
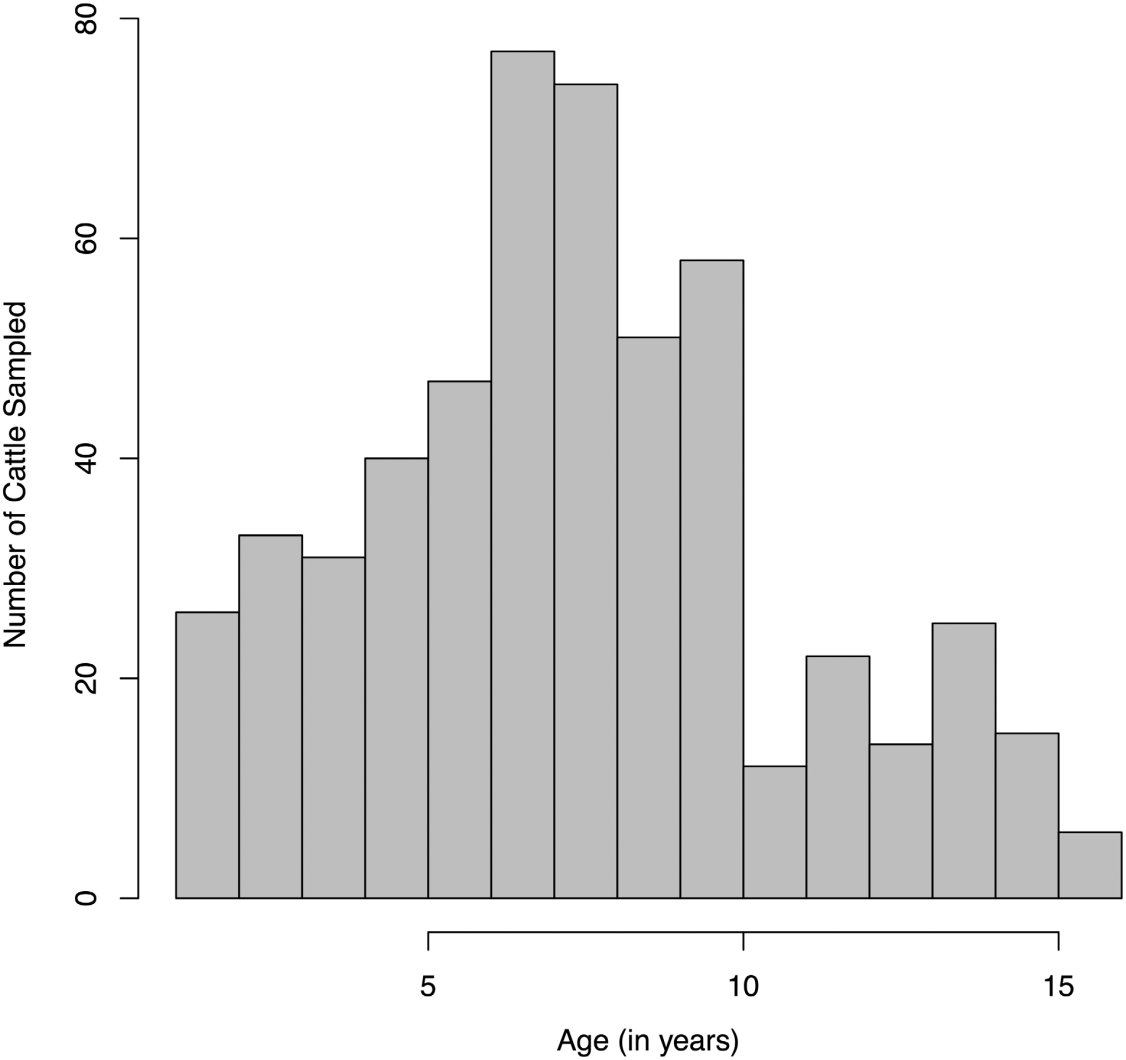
Age distribution of sampled cattle. Four hundred sixty nine cattle, ranging in age from one year old to sixteen years old, were sampled for serotype-specific FMDV antibodies.

Sampled animals ranged in age from one-year-old to sixteen-years-old with a mean of 7.8 years (Fig 1). Ages of animals sampled were similar to previous demographic surveys of cattle in the Far North Region [32]; however, the oldest animals in our study were two years older than the oldest animals previously recorded.

### Serotype-specific waning immunity

As a preliminary investigation to check for any evidence of waning immunity after natural infection with FMDV, we categorized the subset of cattle that were serially sampled cattle (n = 31) and showed seropositivity at the first sampling event and seronegativity at subsequent sampling events. Among the 31 animals that had been sampled twice, seropositivity waned for SAT1 (n = 3), SAT2 (n = 9), SAT3 (n = 4), and type A (n = 4; Table 1). For the single animal that had been sampled three times, seropositivity waned for SAT1, SAT3, and type O. Thus, observations indicate that it is possible that immunity wanes for all five FMDV serotypes but the significance of this process at the population level still needs to be clarified.

**Table 1:** Changes in seropositive status among 31 animals sampled twice in 2010.

FMDV Serotype	Lost	Gained	No change
SAT1	3	1	27
SAT2	9	2	20
SAT3	4	3	24
Type O	0	5	26
Type A	4	5	22

To determine if seropositivity after natural infection was lifelong or waned, we fit the entire dataset consisting of all samples (Table 2) to both the catalytic model (Eq 1) and to the reverse catalytic model (Eq 2).

**Table 2:** Number of samples positive, by age, for all animals sampled in 2010.

Age	SAT1	SAT2	SAT3	A	O	total
1	0	1	0	0	3	8
2	1	6	1	2	8	18
3	8	13	9	15	16	33
4	4	12	2	8	16	31
5	4	13	8	18	29	40
6	8	21	9	15	30	47
7	11	27	14	24	62	77
8	18	37	16	26	54	74
9	18	37	12	22	41	51
10	12	24	13	29	42	58
11	6	6	6	10	10	12
12	8	9	5	9	11	22
13	3	6	2	7	7	14
14	9	10	8	12	16	25
15	3	10	1	7	13	15
16	2	3	0	2	6	6

Model selection, based on AIC scores, indicates if the seropositivity significantly wanes or not at the population level; point estimates for parameter values (*ω*; Eq 2) further quantify the duration of seropositivity. Data for serotypes SAT1 and type O best fit the reverse catalytic model, indicating that seropositivity does not last for the lifespan of cattle in Cameroon (Table 3). Estimated parameters for Type O showed longer duration of seropositivity, lasting for 3.8 years (Table 3). In contrast, estimated parameters for SAT1 showed a shorter duration of seropositivity, inducing antibody reactions that last for less than one year (Table 3). Data for serotypes SAT2, SAT3, and A best fit the catalytic model, indicating that seropostivitiy broadly lasts for the lifespan of cattle in Cameroon (Table 3). Sensitivity of age to model selection results was low, as these results held through analyses in which the animal’s age was increased by 1 or 3 years. In this way, there are serotype-specific host responses that manifest in waning seropositivity for serotypes SAT1 and type O and lifelong seroprevalence for serotypes SAT2, SAT3, and type A.

**Table 3:** Model selection and parameterization.

FMDV Serotype	Model selected	Δ AIC	Duration of natural seropositivity (1/*ω*)	Mean force of infection (*λ*)
SAT1	Reverse catalytic	8.64	0.45 years	0.68 years^-1^
SAT2	Catalytic	6.39	Lifelong	0.021 years^-1^
SAT3	Catalytic	1.53	Lifelong	0.014 years^-1^
Type O	Reverse catalytic	4.02	3.80 years	0.69 years^-1^
Type A	Catalytic	8.29	Lifelong	0.052 years^-1^

### Time-varying serotype-specific FOI

**Fig 2:**
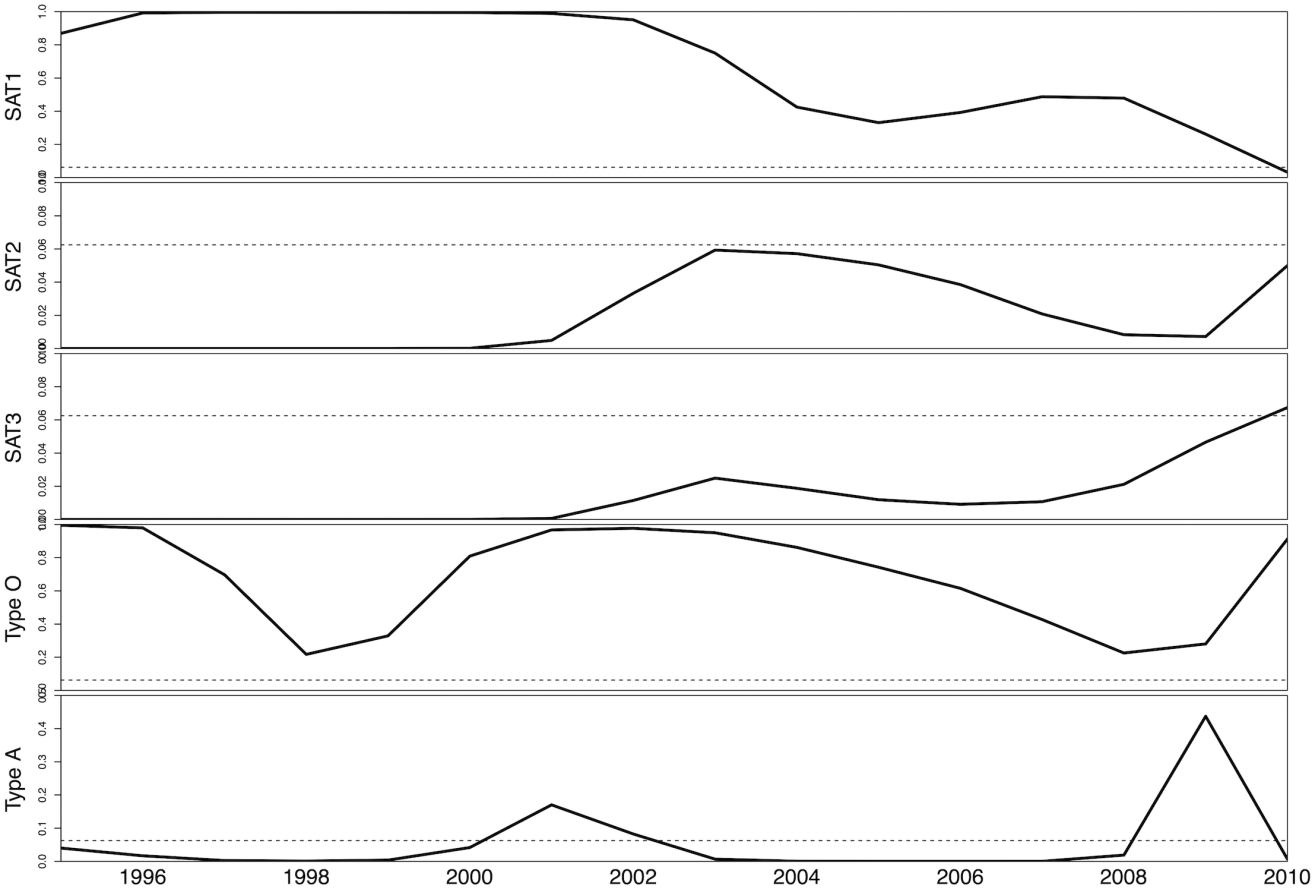
Yearly force of infection by serotype. Estimates derived by model fitting for the time-varying force of infection (FOI) for SAT1, SAT2, SAT2, type O, and type A inferred for the 16-year period preceding data collection represented by a solid line. The dotted line represents the epidemic threshold *R*_0_ = 1; the shaded areas represent estimated FOI that correspond to *R*_0_ > 1 and therefore, sustained chains of transmission within the study population. Note that the scale of the y-axis varies across panels.

**Fig 3:**
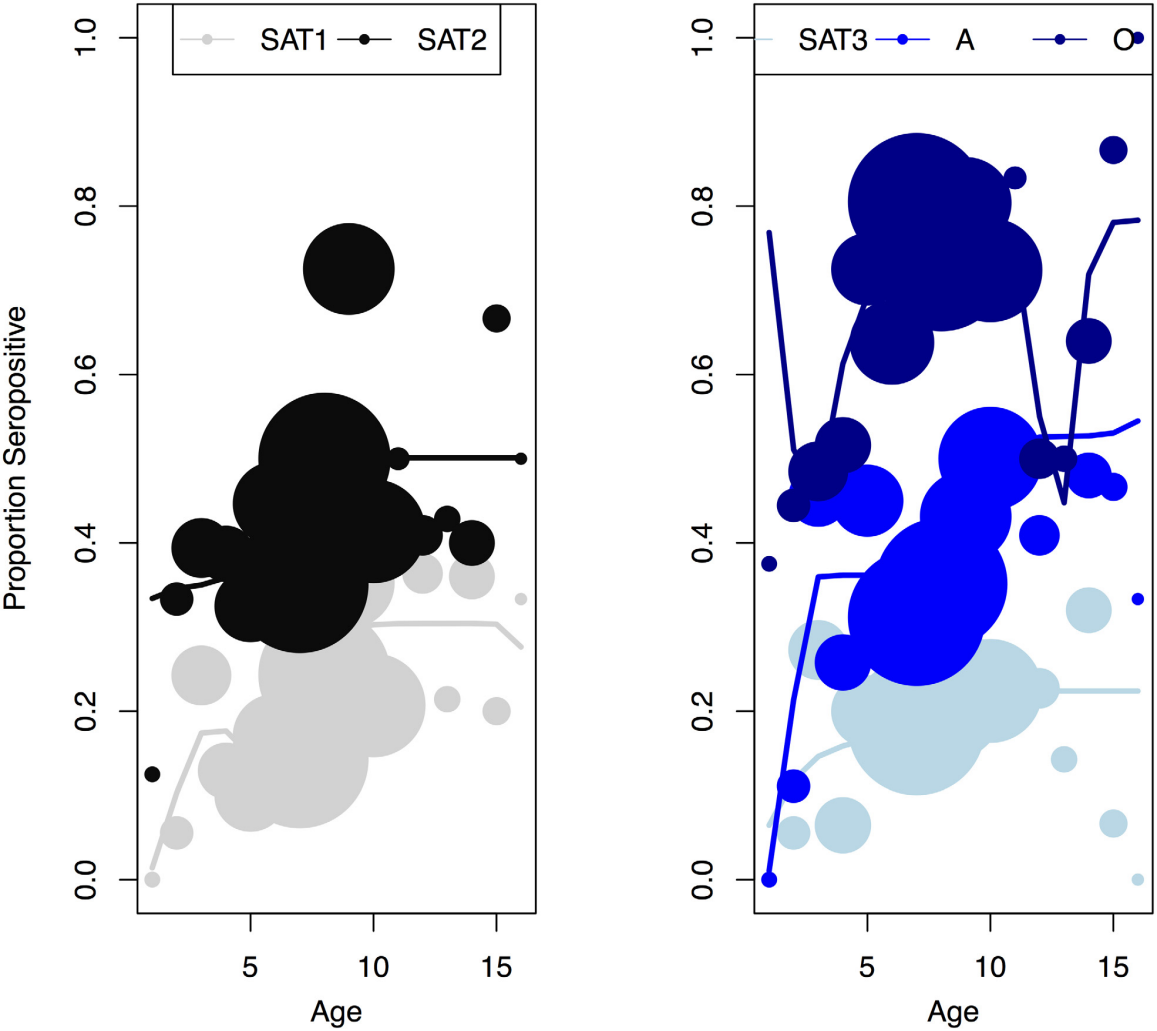
Model selection and parameterization results for five FMDV serotypes. Best-fit model results using the catalytic model (for SAT2, SAT3, and type A) or reverse catalytic mode (for SAT1 and type O) with values for the duration of immunity and serotype-specific time-varying forces of infection estimated from serology data.

To quantify the force of infection, we fit the entire dataset for SAT1 and type O to reverse catalytic models and data for SAT2, SAT3, and A to catalytic models, all of which quantified the time-variation in the force of infection using a b-spline. Since local minima and maxima in the force of infection did not occur at the same ages across serotypes, we infer that the force of infection is time-varying. Between 1996 and 2010, estimates for the FOI for SAT1 decrease through time while estimates for the FOI for SAT2 and SAT3 increase through time (Fig 2). Type O shows cyclic variation in the estimated FOI though time, and type A shows two distinct peaks of estimated FOI: one between 2000 and 2002 and the other between 2008 and 2010. Simulations using the better-fit version of the catalytic model with estimated values for the duration of seropositivity after natural infection and serotype-specific time-varying FOI produced a good fit with the data (Fig 3). The mean estimated FOI ranged from the maximum of 0.69 per year for type O to a minimum of 0.021 per year for SAT 2 (Table 3). Therefore, there are clear serotype specific differences in the FOI with regards to magnitude and temporal patterns.

### Serotype-specific *R*_*t*_ and critical vaccine coverage estimates

To calculate the number of secondary cases produced by each infectious case (*R*_t_), we considered both the mean and the maximum FOI estimated from the best-fit model for each serotype and the cattle lifespan (Eq 4). For SAT1 and type O, *R*_*t*_ could be as high as 16 (Table 4). For SAT2 and SAT3, *R*_*t*_ remained near unity or just below (Table 4). While the average *R*_t_ for type A was below unity, the maximum showed almost 7 secondary cases for each infectious animal. These values resulted in critical proportions (*p*_*c*_) that needed to be vaccinated been 85–95% for SAT1, type O and type A to block circulation (Table 4). Therefore, there were serotype-specific differences in whether or not the estimated FOI breached the epidemic threshold and the targeted vaccination rates to prevent such a breach.

**Table 4:** Epidemiological quantities.

FMDV serotype	mean R_t_	max R_t_	p_c_
SAT1	10.88	15.94	0.94
SAT2	< 1	0.95	n/a
SAT3	< 1	1.08	0.07
Type O	11.04	15.93	0.94
Type A	< 1	6.99	0.86

To determine temporal patterns in epidemic potential, we calculated the FOI that corresponded to the threshold value of *R*_t_ = 1 and plotted it on Fig 2. When the serotype-specific estimated FOI is greater than the threshold value, the population can sustain chains-of-transmission among cattle; when the serotype-specific estimated FOI is less than the threshold value, the population cannot sustain chains-of-transmission. Chains were sustained between 1996 and 2010 by SAT1 and type O and were not sustained by SAT2 and SAT3 (Fig 2). Chains were sustained only for two periods for type A (Fig 2). Thus, there were important time-varying patterns in whether or not the estimated FOI breached the epidemic threshold.

### Variation in transmission and immunity by herd management

To determine if there was variation in the duration of seropositivity or force of infection by herd management type, we stratified the data into three subsets: market cattle, mobile cattle, and sedentary cattle. We fit each subset independently to both the catalytic and reverse catalytic models. We found that the duration of seropositivity was dependent on both serotype and herd management practice: model selection supported waning immunity for 2 serotypes when using the all cattle dataset (Table 3), 1 serotype when using subset of sedentary cattle data, 2 serotypes when using the subset of mobile cattle data, and 4 serotypes when using the subset of market cattle data (A Table in S1 File). Therefore, FMD seroprevalence may be variable and differ among market cattle, mobile cattle, and sedentary cattle.

To determine the serotype-specific FOI, we estimated the parameter from the best-fit model as listed above. We found that the FOI differed between mobile and sedentary cattle and by serotype. For SAT1, SAT2, and SAT3, the FOI was greater in sedentary cattle. For type O and type A, the FOI was greater in mobile cattle (Table 5).

**Table 5:** Mean force of infection (*λ*) for different herd management practices.

FMDV serotype	Mobile cattle	Sedentary cattle
SAT1	0.032 yr^-1^	0.044 yr^-1^
SAT2	0.093 yr^-1^	0.72 yr^-1^
SAT3	0.013 yr^-1^	0.020 yr^-1^
Type O	0.58 yr^-1^	0.19 yr^-1^
Type A	0.60 yr^-1^	0.12 yr^-1^

These results suggest that FMDV transmission differs between mobile and sedentary herds in a complex manner.

### Detection of circulating FMDV serotypes

To determine if changes in circulating FMDV serotypes detected by local surveillance were detected by the models, we quantified the force of infection for 2011 and 2012 by fitting models to samples collected in 2012. For SAT2, the FOI increased over these years, while for serotypes O and A, the FOI decreased. For SAT1 and SAT3, the FOI remained the same over these years. These results correspond with detected increased circulation of SAT2 and decreased circulation of type O between 2010 and 2012 (Table 6).

**Table 6:** Force of infection (*λ*) for all serotypes in 2011 and 2012.

Serotype	2011 FOI	2012 FOI
SAT1	0.0363	0.0336
SAT2	0.0482	0.0655
SAT3	0.0305	0.0305
O	0.127	0.0451
A	9.36 e-6	3.80 e-4

## Discussion

Our results show that the five FMDV serotypes detected in the Far North Region, Cameroon—SAT1, SAT2, SAT3, type O, and type A—differ in their magnitude of transmission as measured by the FOI, duration of host immunity, and their ability to sustain chains-of-transmission. The mean FOI was greatest for serotypes SAT1 and type O, and much lower for serotypes SAT2, SAT3, and type A. Only SAT1 and type O showed waning seropositivity; type O showed a duration of nearly four years while SAT1 seropositivity lasted approximately for half of a year. Time-variation was observed in the FOI, with some serotypes always above the outbreak threshold (SAT1 and type O), other serotypes always below the outbreak threshold (SAT2 and SAT3), and one serotype alternating above and below the threshold (type A). Changes in circulating FMDV serotypes detected by local surveillance in 2011 and 2012 were also detected by our analyses. These results illustrate the complex dynamics that occur in endemic transmission and the numerous differences that exist even between pathogens that are genetically related.

Previous studies investigate the duration of immunity in both experimental and field settings, but show variation in results. Among studies that estimated the duration of immunity in small groups of cattle experimentally infected with FMDV by injection, a single animal was protected against secondary infection 4.5 years after the primary FMDV infection [33] and eight other cattle were protected 5.5 years after the first infection [34], but there was no investigation of immune response at longer durations. On the other hand, a previous survey of cattle in Adamawa Region, Cameroon indicated that antibody prevalence might wane for serotype O [35]. Taken together, these studies indicate no clear pattern for FMDV immunity among cattle. Here, we present two possible explanations for the lack of clear patterns in FMDV immunity. First, the duration of FMDV immunity is serotype specific: some serotypes show lifelong immunity, while others might induce a serological response for less than one year. Second, these results are relative to the lifespan of the host. Waning immunity in an endemic FMDV system where animals have longer lifespans might manifest as lifelong immunity in epidemic FMDV systems where animals have shorter lifespans. In this way, the duration of immunity is specific to both serotype and context of FMDV dynamics.

In Cameroon, where FMDV is endemic, we find periods in which the cattle population can sustain chains-of-transmission internally and other periods in which the cattle population cannot sustain chains-of-transmission but still show a large proportion of seropositive animals. Two serotypes (O and SAT1) show evidence of sustained chains-of-transmission over the last 16 years, indicating an endemic disease regime. One serotype (A) shows periods with chains-of-transmission and other periods without chains-of-transmission, indicating an epidemic disease regime. One period in which our analysis detected sustained chains of transmission for serotype A (2008–2009) corresponds to an outbreak of the same serotype in Nigeria, which borders our study site [36]. Two other serotypes (SAT2 and SAT3) display a lack of chains-of-transmission, indicating that transmission occurs via stuttering chains or repeated contact with some other animal reservoir. The disease regime affects control strategies, and vaccinating 85–95% of the population would help control serotypes that display endemic or epidemic behavior. However, for those serotypes that exhibit other disease mechanisms, controlling access to a source population may be more beneficial. These results indicate that complex dynamics govern endemic FMD and must be considered when planning appropriate control programs.

Certain features of the analysis permitted the characterization of serotype specific epidemiological parameters and dynamics. First, the comparison of multiple pathogens sampled from the same animals at the same time allowed the FOI to be categorized as either age-specific or time-varying. Because the FOI did not show the same patterns with age—the maximum or minimum values did not occur at the same ages across serotypes—then we can conclude that the FOI must be time-varying. Because age-specificity in the FOI has been well-documented across pathogens, it is likely that there are also some age-specific factors at play, including between-host contacts or within-host immunological responses that differ by age. Second, the data collection scheme permitted separation of two fundamental phenomena that drive transmission: pathogen characteristics and patterns of contact within a population. These samples were obtained from the same animals at the same time, effectively holding patterns of contact constant. Factors uncontrolled for in this analysis would have affected all serotypes equally, since identical animals were sampled for all five serotypes. Any differences among the serotypes can be attributed to differences in pathogen characteristics that are specific to each serotype. Thus, time-variation and serotype-specific differences driven by viral biology can be detected.

Serology from serially sampled animals did not show waning antibody prevalence for serotype O; however, the model selected indicated that immunity lasted for almost four years and then waned. This combination might arise for pathogens that display cyclic multiannual dynamics sampled between the onset of the outbreak and the peak number of cases. If our sampling occurred as an outbreak was in an early phase, most animals would be gaining immunity and no information about waning immunity or immune duration would be provided by only analyzing paired samples. It would be interesting to sample serology over multiple years and during different seasons to determine if the signature of cyclic outbreaks or waning immunity could be detected.

Serology from serially sampled animals indicated waning antibody prevalence for serotypes SAT2, SAT3, and type A; however, the model selected indicated lifelong antibody prevalence. This discrepancy might arise if the duration of antibody prevalence is so short that the waning cannot be detected in models that have a timestep of one year. The serotypes that show the discrepancy are those with the highest counts of immunity loss in the paired sample data, so it is possible that they exhibit waning immunity. It would be interesting to sample serology at shorter intervals over multiple years and fit catalytic and reverse catalytic models with shorter timesteps to see if waning immunity can be detected at an enhanced temporal resolution.

Our analyses suggested variation in the FOI and duration of immunity among market cattle, mobile cattle, and sedentary cattle. Variation might be caused by greater stress in market and mobile herds, which has been shown to negatively impact immune function. Alternatively, variation might be driven by heterogeneity in disease exposures that differ between herd types, with repeated exposure in sedentary cattle causing immune boosting that lengthens immune durations for multiple serotypes. In either case, if control were to be implemented in Cameroon, mobile and market herds would be ideal candidates for vaccination since waning immunity suggested for multiple serotypes.

## Conclusion

FMDV endemicity is complex due to the presence of multiple co-circulating serotypes that differ in characteristics relating to transmission and immunity. Although epidemic patterns and processes are well defined [37–41], they do not accurately represent the true global FMD burden, which occurs primarily in endemic settings [42, 43]. In order to understand the epidemiology of this pathogen, we must consider patterns and processes in infection and immunity that differ from the well-studied patterns evident in acute, immunizing diseases.

## Supporting Information

S1 File Supplementary Material for Serotype-specific transmission and waning immunity of endemic foot-and-mouth disease virus including dates of serology sampling (**A Text** and **Fig A**), model derivation (**B Text**), derivation for age-specific FOI versus time-varying FOI (**C Text**), additional results from stratifying data by herd management type (**D Text** and **Table A**), and additional results from analyzing 2012 serology data (**E Text** and **Table B**).
(PDF)
