# Supplementary material for: Serotype-Specific Transmission and Waning Immunity of Endemic Foot-and-Mouth Disease Virus in Cameroon
Source: PLoS One. 2015 Sep 1;10(9):e0136642. doi: 10.1371/journal.pone.0136642 (PMC4556668; doi:10.1371/journal.pone.0136642)
Supplement: S1 File — Supplementary Material for Serotype-specific transmission and waning immunity of endemic foot-and-mouth disease virus including dates of serology sampling (A Text and Fig A), model derivation (B Text), derivation for age-specific FOI versus time-varying FOI (C Text), additional results from stratifying data by herd management type (D Text and Table A), and additional results from analyzing 2012 serology data (E Text and Table B). (PDF) [file pone.0136642.s001.pdf]

Supplementary Material for Serotype-specific transmission and waning immunity of endemic foot-and-mouth disease virus

Laura W. Pomeroy, Ottar N. Bjørnstad, Hyeyoung Kim, Simon Dickmu Jumbo, Souley Abdoulkadiri, Rebecca B. Garabed

correspondence to: **pomeroy.26@osu.edu**

A. Dates of serology sampling

Between February 5, 2010 and December 29, 2010, 498 animals were sampled in the Far North Region Cameroon (Figure A). The animals that were sampled once (n=466) were sampled at dates throughout the study period. The animals sampled twice (n=31) were sampled for the first time between February 6 and March 27; the same animals were sampled for the second time between June 23 and August 16. The animal that was sampled three times was sampled twice in February (February 16 and February 26) and once one June 30.

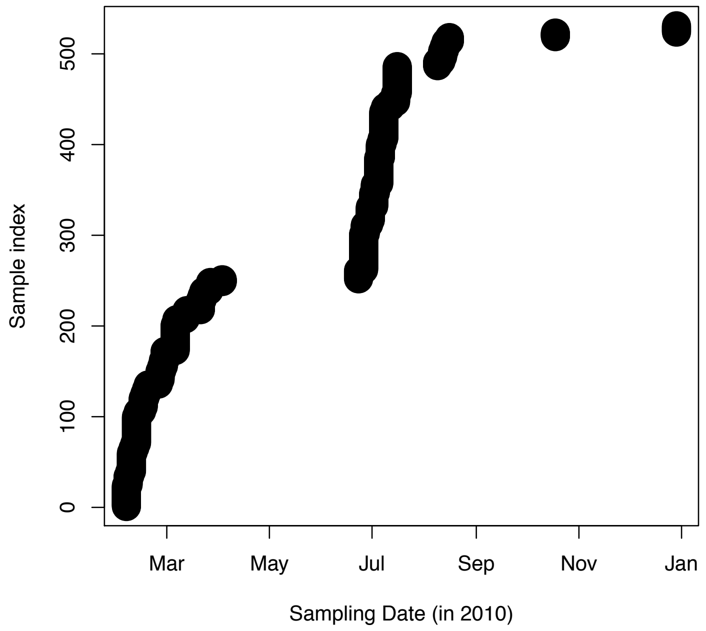

Figure A: Dates of sampling for 498 animals in the Far North Region, Cameroon.

B. Model derivation

Consider a two-state disease system, in which individuals are classified relative to their disease status. Let S and P represent proportions of the total population, such that $S + P = 1$.

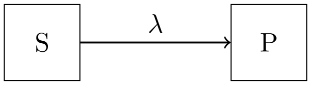

Figure B: Catalytic model. *Susceptible individuals* (S) test negative for disease and are susceptible to contracting it. *Positive individuals* (P) show evidence of previous or current infection. The force of infection, or λ, is the rate at which individuals acquire disease and convert from susceptible to positive disease status.

For the catalytic model, and assuming an age-specific force of infection (FOI) represented by $\lambda(a)$,

$$\frac{dP(a)}{da} = \lambda(a)(1 - P(a)). \tag{1}$$

Solving for $P(a)$ gives

$$P(a) = 1 + Ce^{-\int_0^a \lambda(a)da}. \tag{2}$$

Assume that all individuals are susceptible at birth such that $P(a) = 0$ when $a = 0$. Then, $C = -1$ and

$$P(a) = 1 - e^{-\int_0^a \lambda(a)da}. \tag{3}$$

The catalytic models above assume lifelong immunity implying that conversion to the seropostive state is permanent and individuals cannot convert back to the seronegative state. This may or may not be a valid assumption for FMDV serostatus. The reversible catalytic model relaxes the assumption of lifelong immunity, so that the duration of immunity may be considered at variable intervals. Again, let S and P represent proportions of the total population, such that $S + P = 1$.

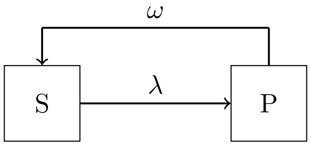

Figure C: Reversible catalytic model. *Susceptible individuals* (S) test negative for disease and are susceptible to contracting it. *Positive individuals* (P) show evidence of previous or current infection. The force of infection, or λ, is the rate at which individuals acquire disease and convert from negative to positive disease status and the rate of waning immunity, or ω, permits re-entry into the seronegative serostatus.

For the reversible catalytic model, again assuming an age-specific FOI represented by $\lambda(a)$,

$$\frac{dP(a)}{da} = \lambda(a)(1 - P(a)) - \omega P(a). \tag{4}$$

Solving for $P(a)$,

$$P(a) = \frac{\lambda(a)}{\lambda(a) + \omega} + Ce^{-\int_0^a (\lambda(a)+\omega)da} \tag{5}$$

Again, assume that all individuals are susceptible at birth such that $P(a) = 0$ when $a = 0$. Then, $C = -\frac{\lambda(a)}{\lambda(a)+\omega}$ and

$$P(a) = \frac{\lambda(a)}{\lambda(a) + \omega}(1 - e^{-\int_0^a (\lambda(a)+\omega)da}). \quad (6)$$

C. Age-specific FOI versus time-varying FOI

Let I represent the number of infectious animals and let N represent the total population size. Consider that the FOI (λ) depends on the rate of contact between animals (c), the probability that contact will be with an infectious individual ($\frac{I}{N}$), and the probability that contact with an infectious individual produces a new infection (p) such that

$$\lambda = c\frac{I}{N}p \quad (7)$$

following Begon (2002). Previous work has indicated that λ can vary with age and/or with time, such that the force of infection for the ith serotype can be written as

$$\lambda_i(a, t) = c(a)\frac{I_i(t)}{N}p. \quad (8)$$

If the variation in λ_i is due to age-related variation in the contact rate, then the age(s) at which each serotype exhibits maximum and minimum λ_i occur(s) at

$$\frac{d\lambda_i(a)}{da} = \frac{dc(a)}{da} = 0. \quad (9)$$

Given an age-specific λ_i, the maximum and minimum would occur at the same ages regardless of serotypes.

If the variation in λ_i is due to time-varying abundance of infectious individuals, then setting

$$\frac{d\lambda_i(t)}{dt} = \frac{dI_i(t)}{dt} = 0 \quad (10)$$

would result in a curve for λ_i in which the age at which each serotype exhibits maximum and minimum λ_i varies by serotype, assuming asynchrony in $I_i(t)$

D. Additional results from stratifying data by herd management type

We stratified the dataset by herd management type so that animals in mobile herds, sedentary herds, and markets were considered as three separate groups. We then fit each data subset to the catalytic and reverse catalytic models. Model selection results are found in the table below.

FMDV serotype	All cattle	Market Cattle	Mobile Cattle	Sedentary Cattle
SAT1	waning	waning	lifelong	lifelong
SAT2	lifelong	lifelong	lifelong	waning
SAT3	lifelong	waning	lifelong	lifelong
Type O	waning	waning	waning	lifelong
Type A	lifelong	waning	waning	lifelong

Table A: Duration of immunity suggested by model selection.

E. Additional results from analyzing 2012 serology data

A small number of samples (n=124) were available from 2012. Serology data are listed in the table below.

Age	SAT1	SAT2	SAT3	Type O	Type A	Total (N)
1	2	3	0	0	1	10
2	3	7	1	7	3	20
3	2	7	1	7	2	13
4	3	12	0	7	6	16
5	3	7	0	5	5	10
6	3	3	0	7	2	9
7	1	10	1	6	3	15
8	1	10	2	10	7	13
9	1	5	1	5	5	7
10	0	3	0	4	4	6
11	1	3	0	3	0	3
12	0	1	0	0	0	2

Table B. Number of samples positive, by age, for all animals sampled in 2012.

The catalytic model was selected by AIC for all five serotypes when fitting the model only with data from 2012.

F. Reference

Begon, M and Bennett, M and Bowers, R G and French, N P and Hazel, S M and Turner, J. 2002. A clarification of transmission terms in host-microparasite models: numbers, densities and areas. *Epidemiology and Infection*, 129(1), pages 147-153.
